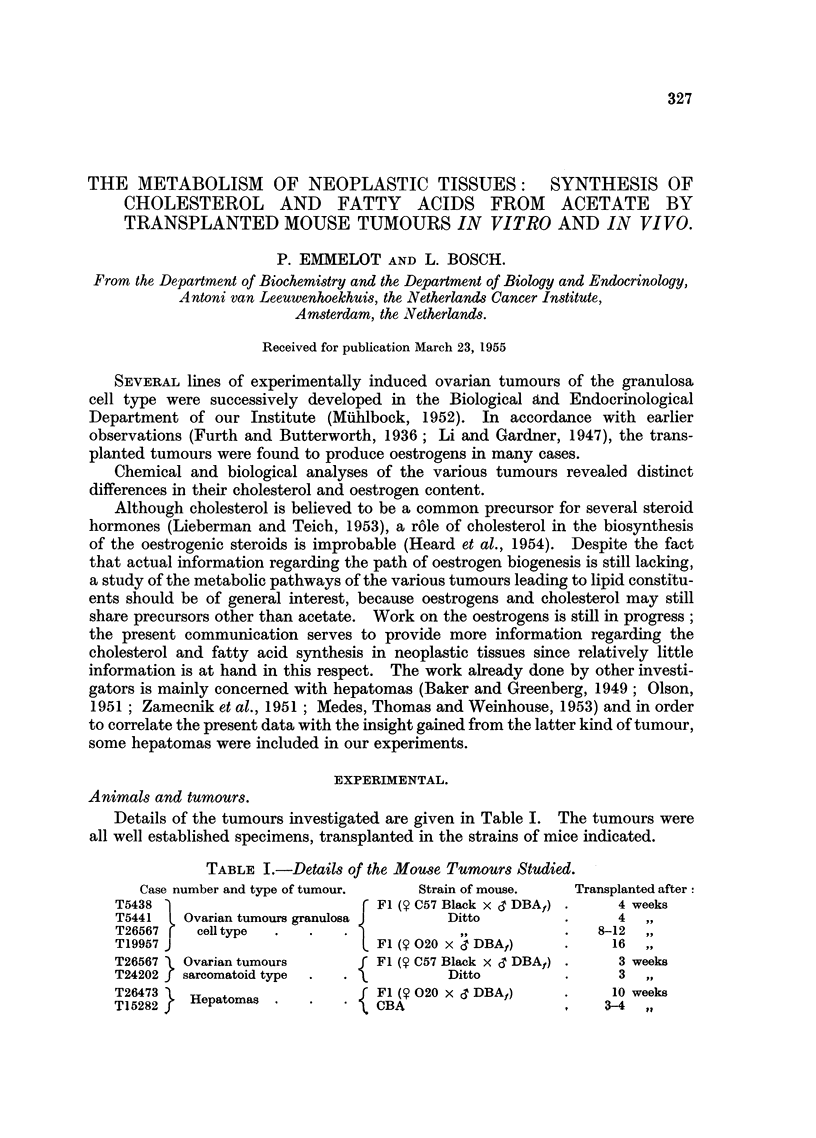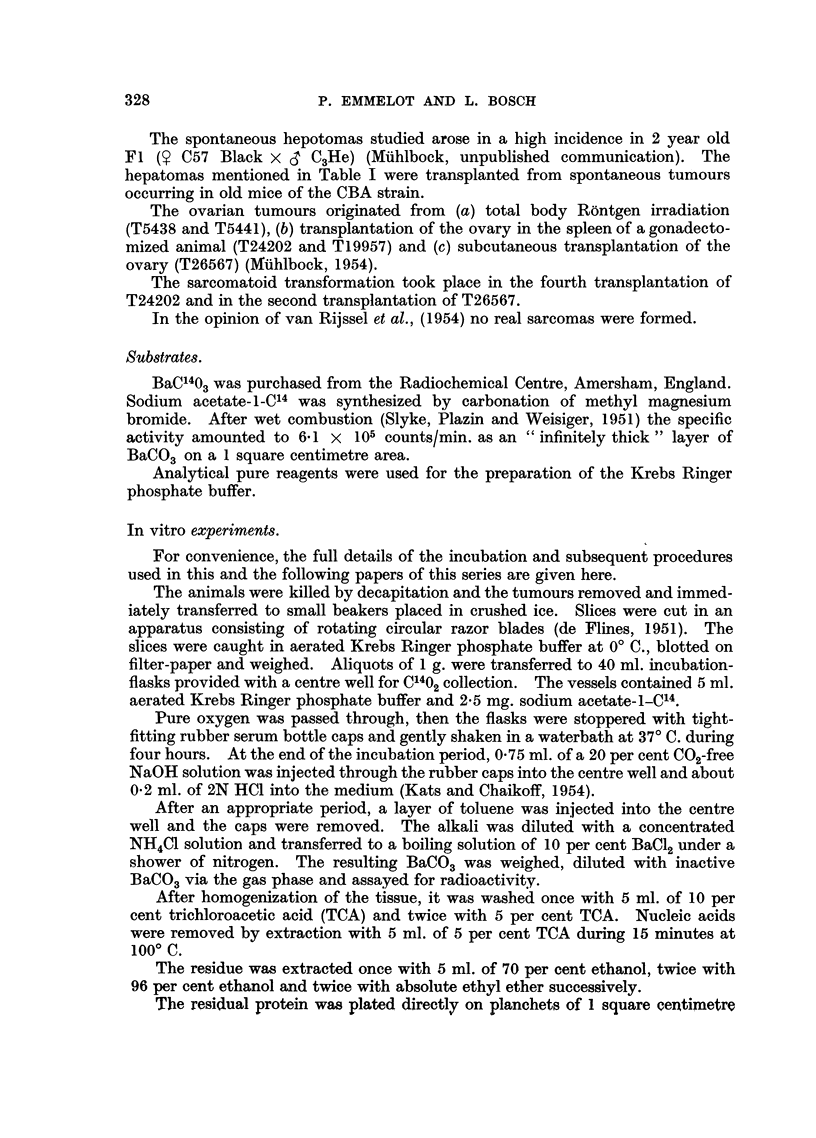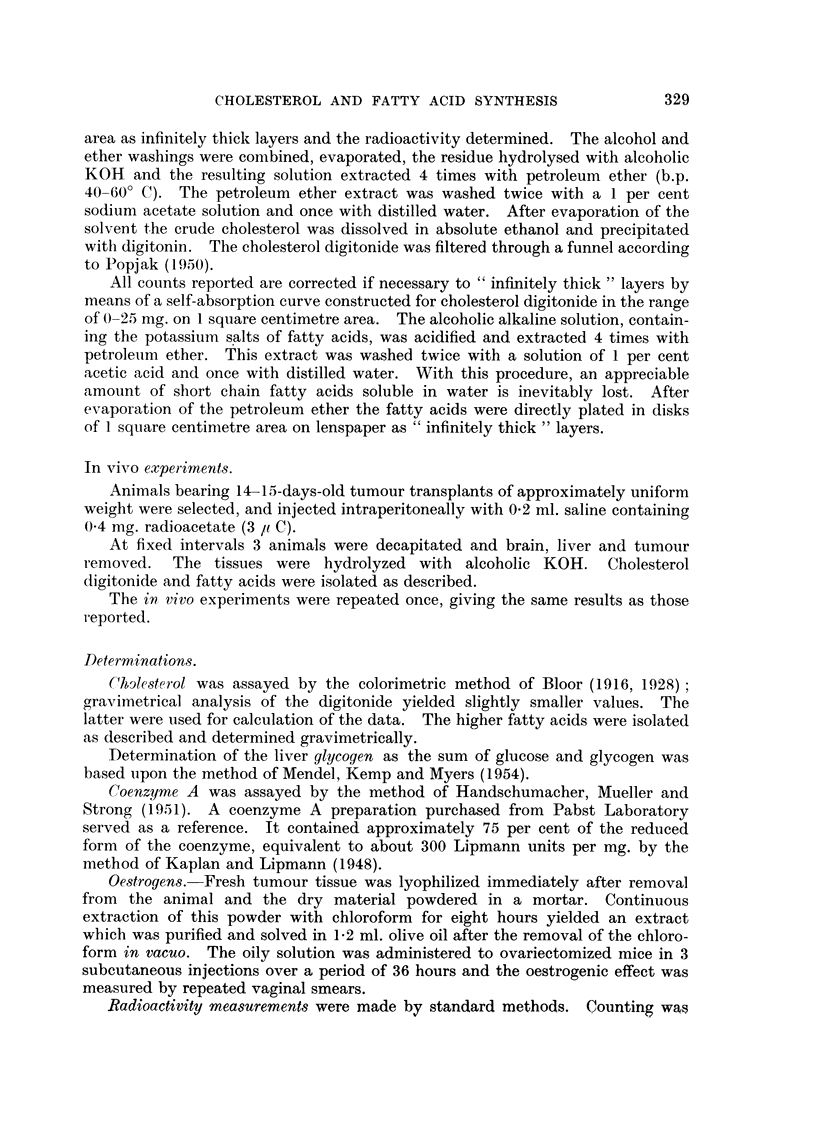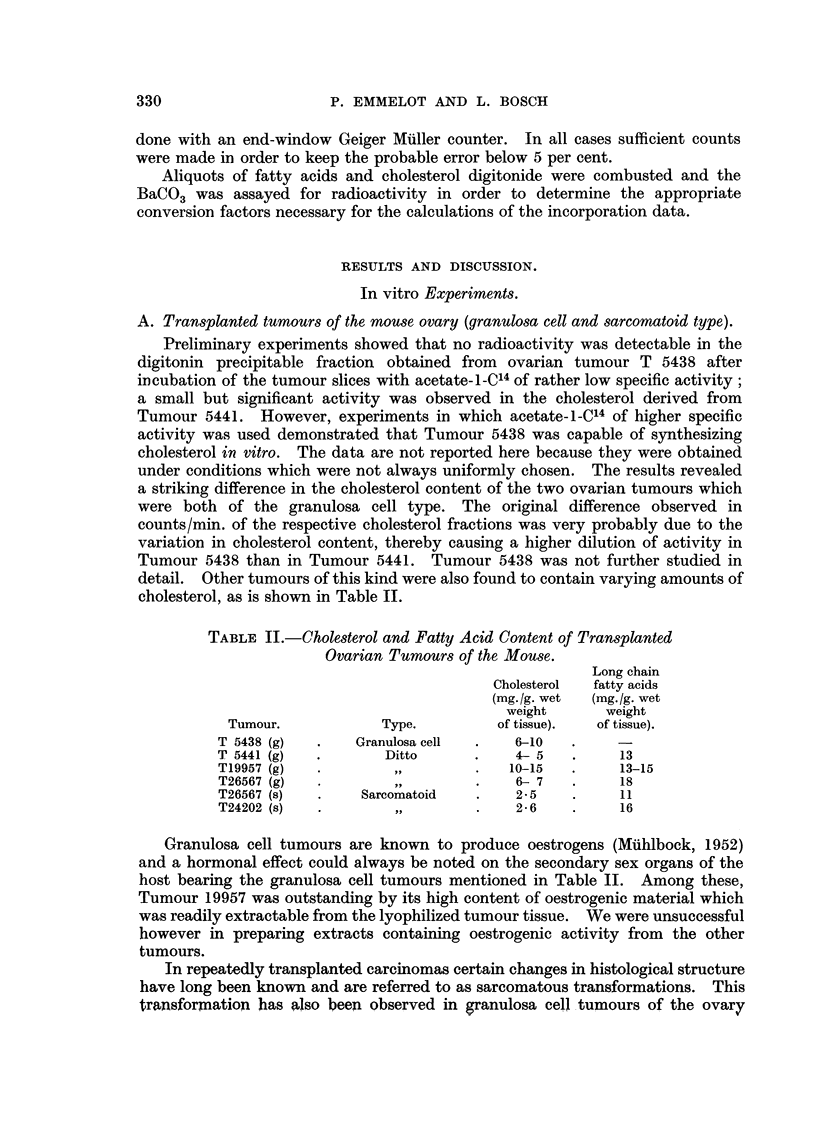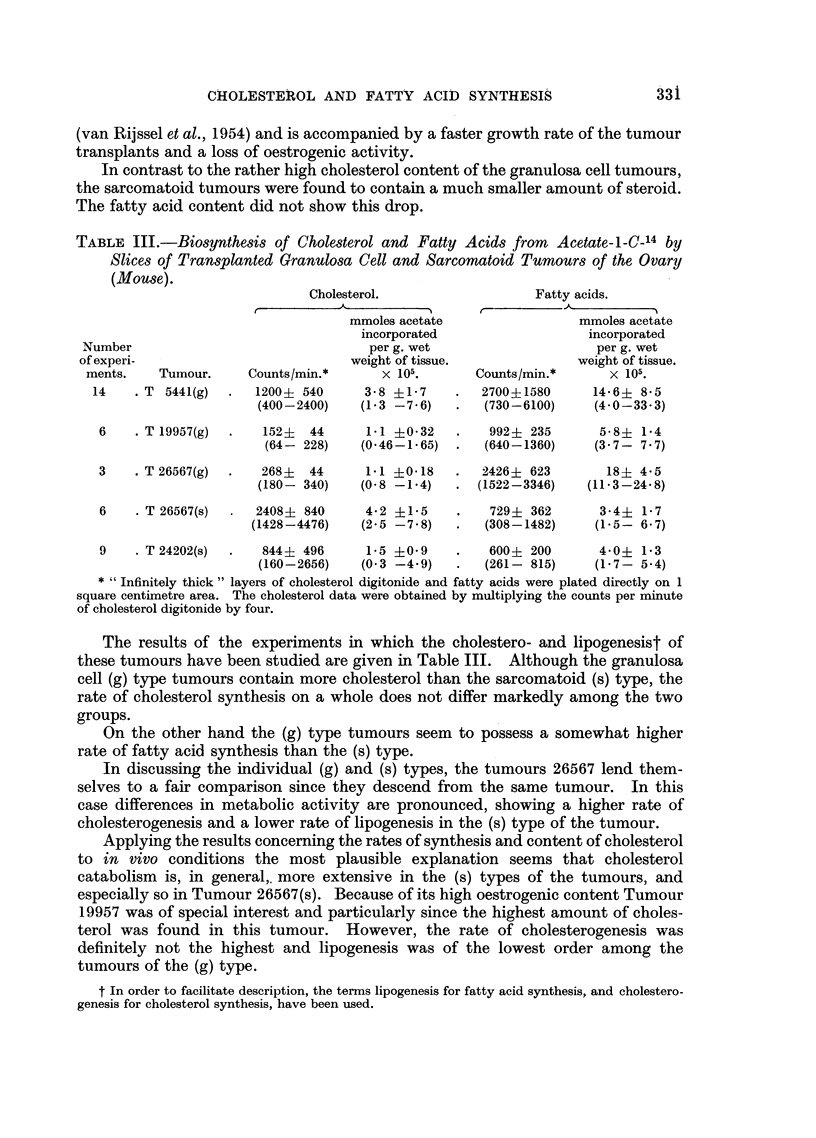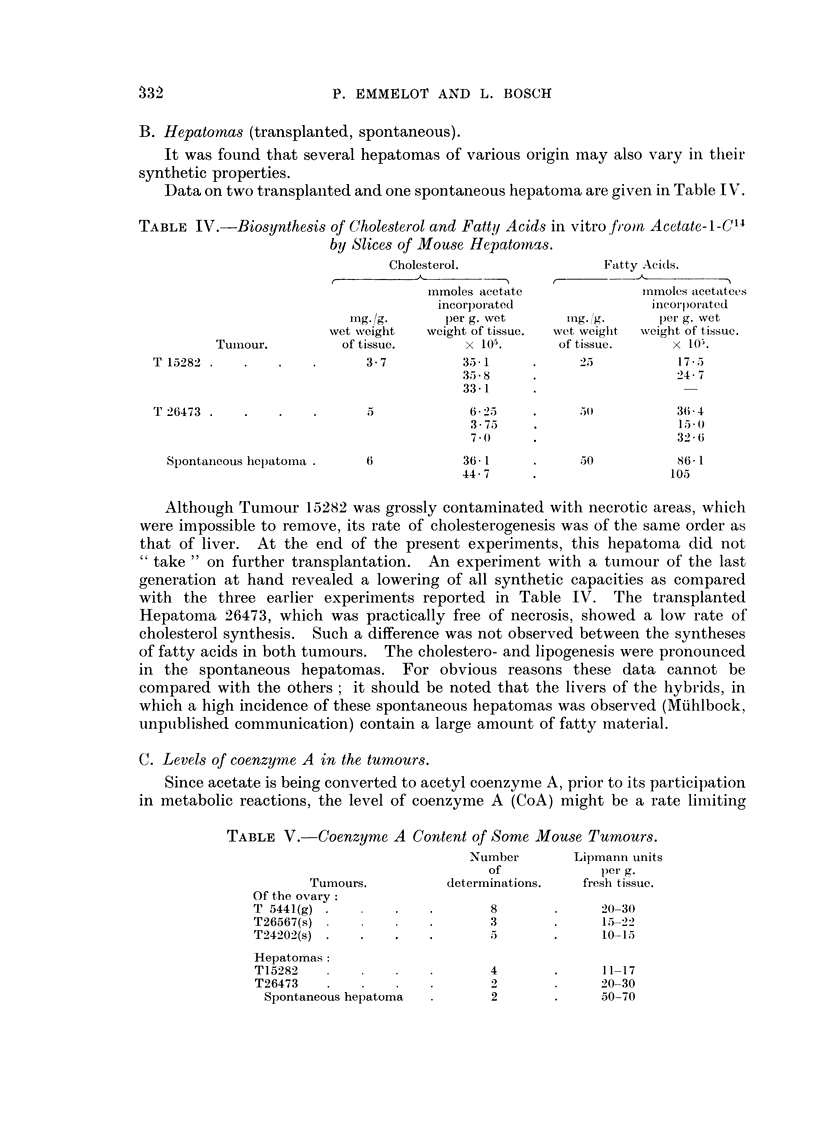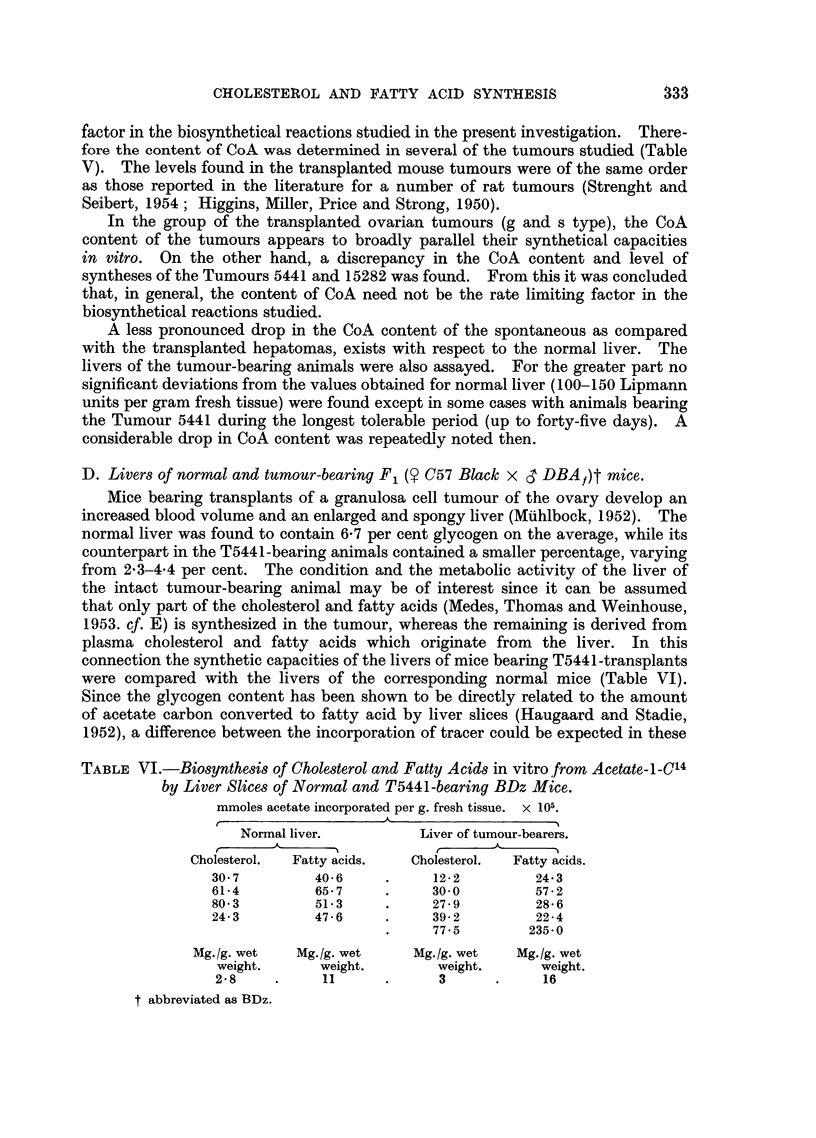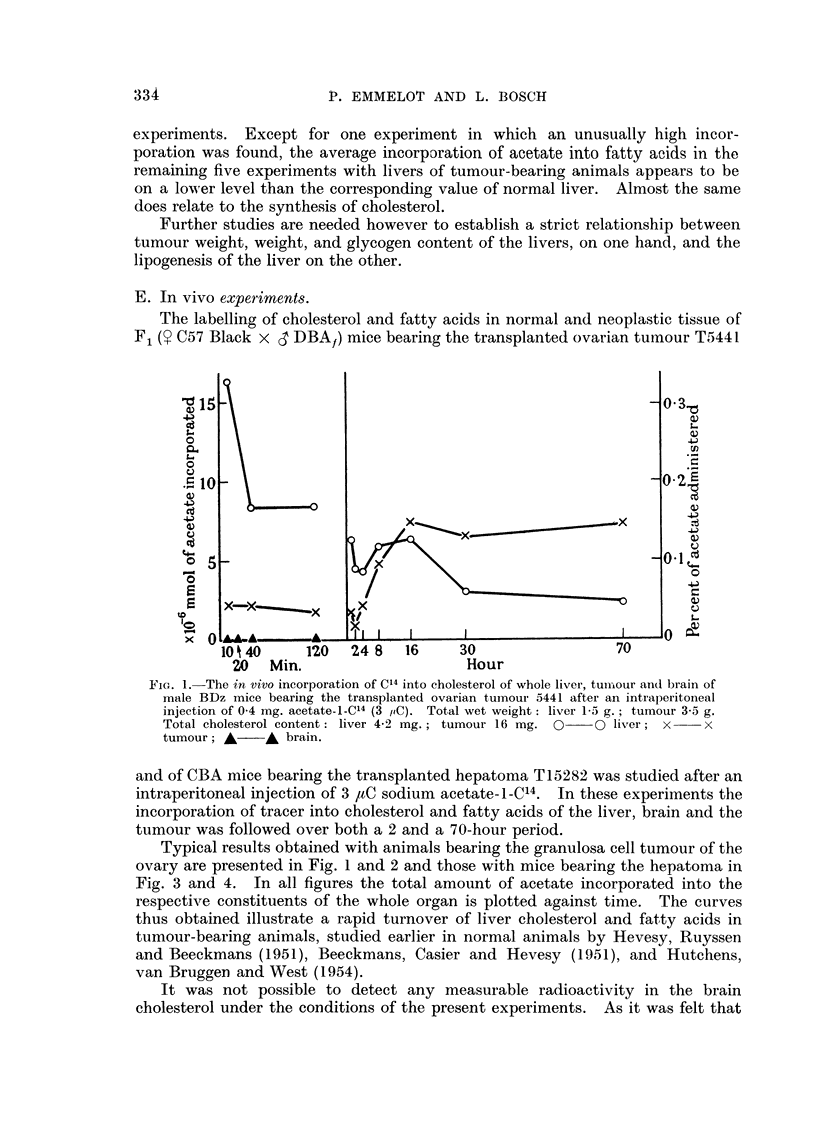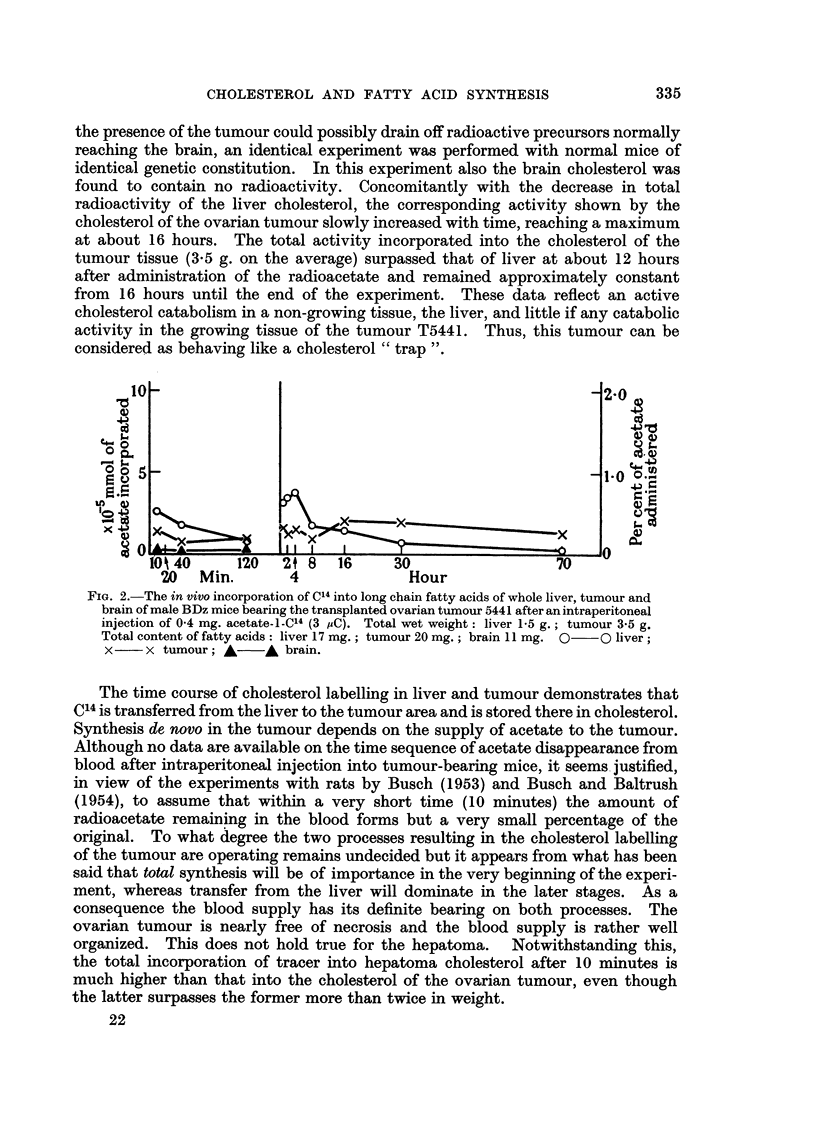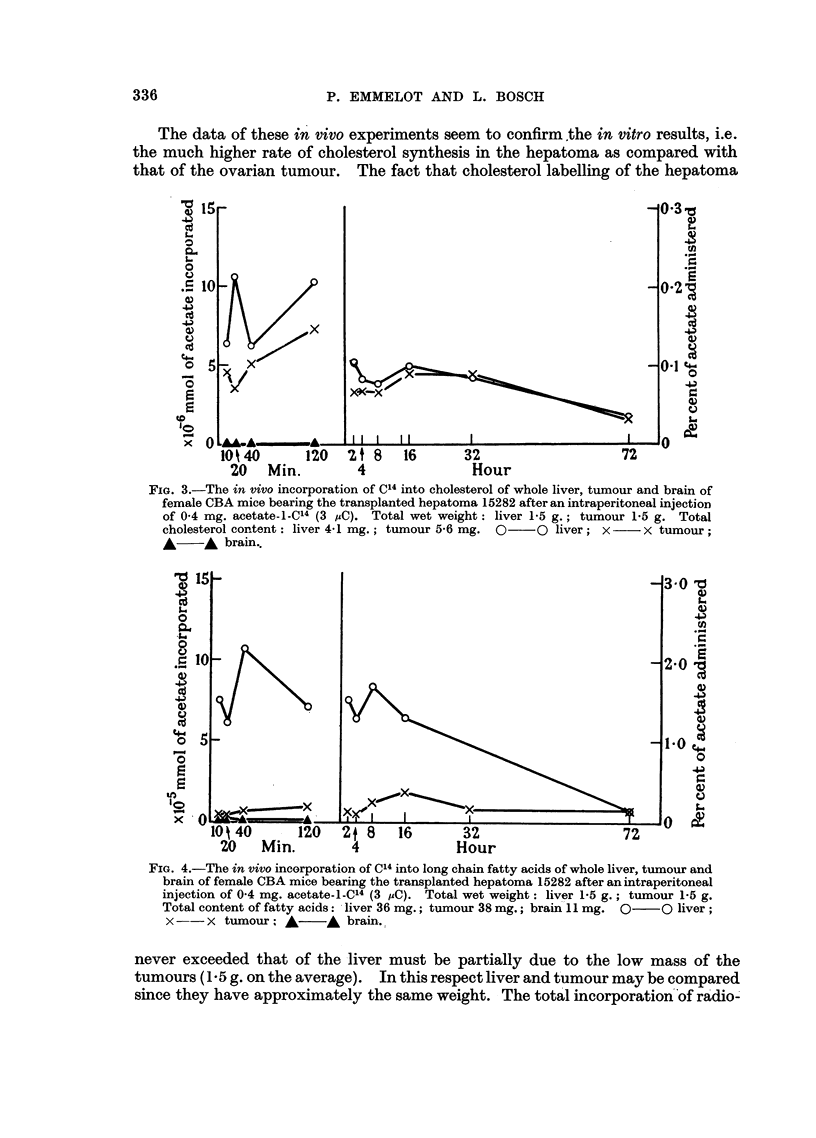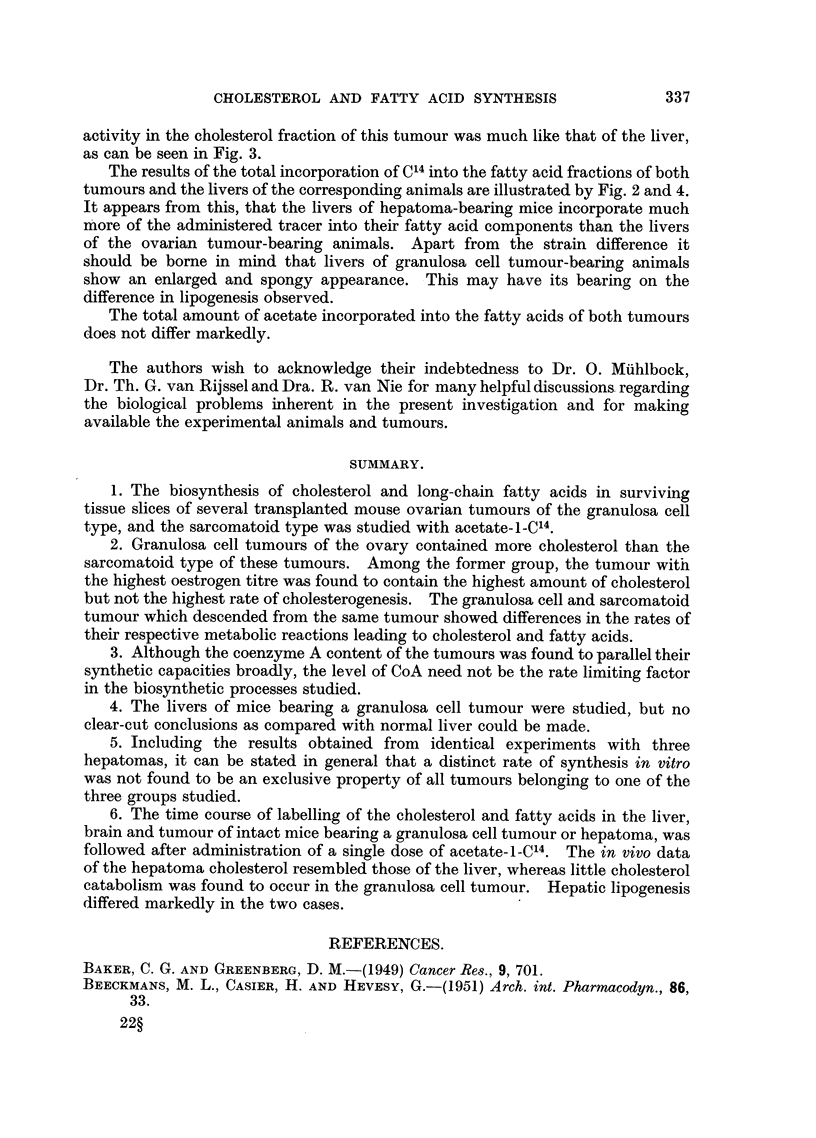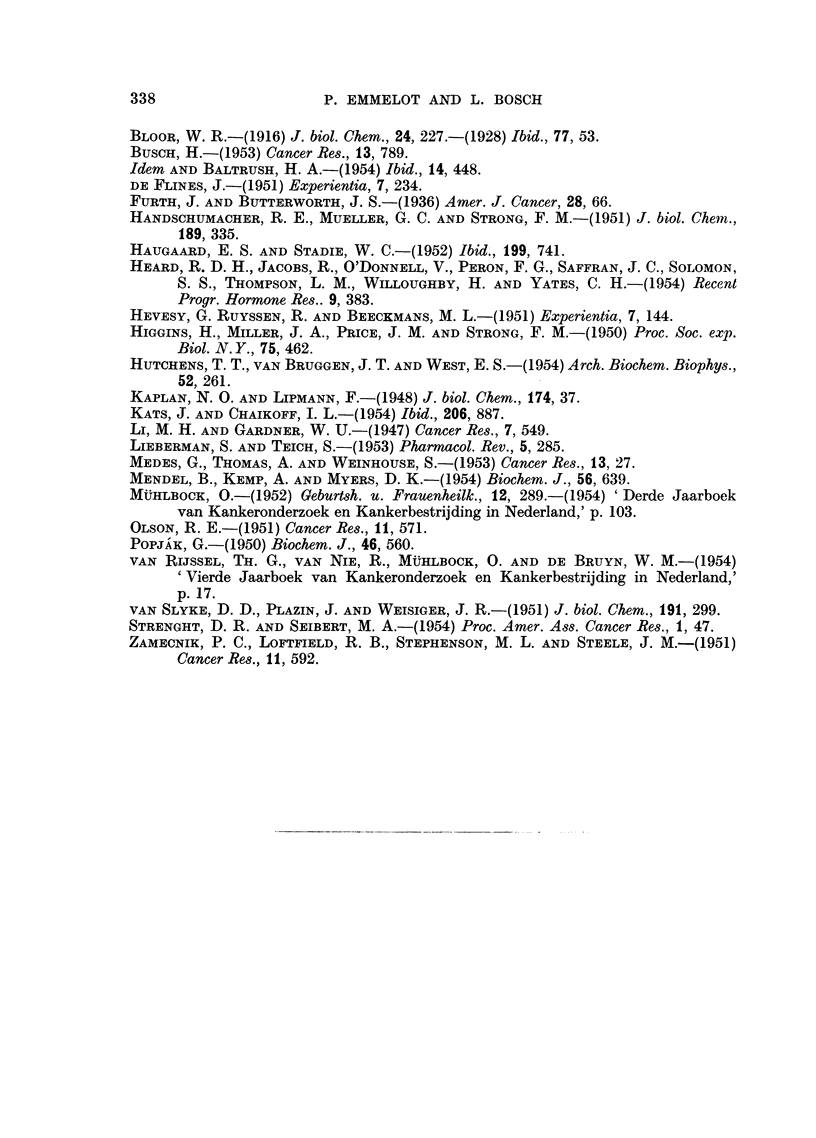# The Metabolism of Neoplastic Tissues: Synthesis of Cholesterol and Fatty Acids from Acetate by Transplanted Mouse Tumours in Vitro and in Vivo

**DOI:** 10.1038/bjc.1955.30

**Published:** 1955-06

**Authors:** P. Emmelot, L. Bosch


### THE METABOLISM OF NEOPLASTIC TISSUES: SYNTHESIS OF CHOLESTEROL AND FATTY ACIDS FROM ACETATE BY TRANSPLANTED MOUSE TUMOURS *IN VITRO* AND *IN VIVO*.

P. EMMELOT AND L. BOSCH.

*From the Department of Biochemistry and the Department of Biology and Endocrinology, Antoni van Leeuwenhoekhuis, the Netherlands Cancer Institute, Amsterdam, the Netherlands.*

Received for publication March 23, 1955

SEVERAL lines of experimentally induced ovarian tumours of the granulosa cell type were successively developed in the Biological and Endocrinological Department of our Institute (Mühlbock, 1952). In accordance with earlier observations (Furth and Butterworth, 1936; Li and Gardner, 1947), the transplanted tumours were found to produce oestrogens in many cases.

Chemical and biological analyses of the various tumours revealed distinct differences in their cholesterol and oestrogen content.

Although cholesterol is believed to be a common precursor for several steroid hormones (Lieberman and Teich, 1953), a rôle of cholesterol in the biosynthesis of the oestrogenic steroids is improbable (Heard *et al.*, 1954). Despite the fact that actual information regarding the path of oestrogen biogenesis is still lacking, a study of the metabolic pathways of the various tumours leading to lipid constituents should be of general interest, because oestrogens and cholesterol may still share precursors other than acetate. Work on the oestrogens is still in progress; the present communication serves to provide more information regarding the cholesterol and fatty acid synthesis in neoplastic tissues since relatively little information is at hand in this respect. The work already done by other investigators is mainly concerned with hepatomas (Baker and Greenberg, 1949; Olson, 1951; Zamecnik *et al.*, 1951; Medes, Thomas and Weinhouse, 1953) and in order to correlate the present data with the insight gained from the latter kind of tumour, some hepatomas were included in our experiments.

### EXPERIMENTAL.

### *Animals and tumours.*

Details of the tumours investigated are given in Table I. The tumours were all well established specimens, transplanted in the strains of mice indicated.

TABLE I.—*Details of the Mouse Tumours Studied.*

| Case number | Type of tumour | Strain of mouse | Transplanted after: |
|---|---|---|---|
| T5438 | Ovarian tumours granulosa cell type | F1 (♀ C57 Black × ♂ DBA$_f$) | 4 weeks |
| T5441 | | Ditto | 4 ,, |
| T26567 | | ,, | 8–12 ,, |
| T19957 | | F1 (♀ O20 × ♂ DBA$_f$) | 16 ,, |
| T26567 | Ovarian tumours sarcomatoid type | F1 (♀ C57 Black × ♂ DBA$_f$) | 3 weeks |
| T24202 | | Ditto | 3 ,, |
| T26473 | Hepatomas | F1 (♀ O20 × ♂ DBA$_f$) | 10 weeks |
| T15282 | | CBA | 3–4 ,, |

The spontaneous hepotomas studied arose in a high incidence in 2 year old F1 (♀ C57 Black × ♂ $C_3He$) (Mühlbock, unpublished communication). The hepatomas mentioned in Table I were transplanted from spontaneous tumours occurring in old mice of the CBA strain.

The ovarian tumours originated from (*a*) total body Röntgen irradiation (T5438 and T5441), (*b*) transplantation of the ovary in the spleen of a gonadectomized animal (T24202 and T19957) and (*c*) subcutaneous transplantation of the ovary (T26567) (Mühlbock, 1954).

The sarcomatoid transformation took place in the fourth transplantation of T24202 and in the second transplantation of T26567.

In the opinion of van Rijssel *et al.*, (1954) no real sarcomas were formed.

*Substrates.*

$BaC^{14}0_3$ was purchased from the Radiochemical Centre, Amersham, England. Sodium acetate-1-$C^{14}$ was synthesized by carbonation of methyl magnesium bromide. After wet combustion (Slyke, Plazin and Weisiger, 1951) the specific activity amounted to $6{\cdot}1 \times 10^5$ counts/min. as an " infinitely thick " layer of $BaCO_3$ on a 1 square centimetre area.

Analytical pure reagents were used for the preparation of the Krebs Ringer phosphate buffer.

In vitro *experiments.*

For convenience, the full details of the incubation and subsequent procedures used in this and the following papers of this series are given here.

The animals were killed by decapitation and the tumours removed and immediately transferred to small beakers placed in crushed ice. Slices were cut in an apparatus consisting of rotating circular razor blades (de Flines, 1951). The slices were caught in aerated Krebs Ringer phosphate buffer at 0° C., blotted on filter-paper and weighed. Aliquots of 1 g. were transferred to 40 ml. incubation-flasks provided with a centre well for $C^{14}0_2$ collection. The vessels contained 5 ml. aerated Krebs Ringer phosphate buffer and 2·5 mg. sodium acetate-1–$C^{14}$.

Pure oxygen was passed through, then the flasks were stoppered with tight-fitting rubber serum bottle caps and gently shaken in a waterbath at 37° C. during four hours. At the end of the incubation period, 0·75 ml. of a 20 per cent $CO_2$-free NaOH solution was injected through the rubber caps into the centre well and about 0·2 ml. of 2N HCl into the medium (Kats and Chaikoff, 1954).

After an appropriate period, a layer of toluene was injected into the centre well and the caps were removed. The alkali was diluted with a concentrated $NH_4Cl$ solution and transferred to a boiling solution of 10 per cent $BaCl_2$ under a shower of nitrogen. The resulting $BaCO_3$ was weighed, diluted with inactive $BaCO_3$ via the gas phase and assayed for radioactivity.

After homogenization of the tissue, it was washed once with 5 ml. of 10 per cent trichloroacetic acid (TCA) and twice with 5 per cent TCA. Nucleic acids were removed by extraction with 5 ml. of 5 per cent TCA during 15 minutes at 100° C.

The residue was extracted once with 5 ml. of 70 per cent ethanol, twice with 96 per cent ethanol and twice with absolute ethyl ether successively.

The residual protein was plated directly on planchets of 1 square centimetre area as infinitely thick layers and the radioactivity determined. The alcohol and ether washings were combined, evaporated, the residue hydrolysed with alcoholic KOH and the resulting solution extracted 4 times with petroleum ether (b.p. 40–60° C). The petroleum ether extract was washed twice with a 1 per cent sodium acetate solution and once with distilled water. After evaporation of the solvent the crude cholesterol was dissolved in absolute ethanol and precipitated with digitonin. The cholesterol digitonide was filtered through a funnel according to Popjak (1950).

All counts reported are corrected if necessary to " infinitely thick " layers by means of a self-absorption curve constructed for cholesterol digitonide in the range of 0–25 mg. on 1 square centimetre area. The alcoholic alkaline solution, containing the potassium salts of fatty acids, was acidified and extracted 4 times with petroleum ether. This extract was washed twice with a solution of 1 per cent acetic acid and once with distilled water. With this procedure, an appreciable amount of short chain fatty acids soluble in water is inevitably lost. After evaporation of the petroleum ether the fatty acids were directly plated in disks of 1 square centimetre area on lenspaper as " infinitely thick " layers.

In vivo *experiments.*

Animals bearing 14–15-days-old tumour transplants of approximately uniform weight were selected, and injected intraperitoneally with 0·2 ml. saline containing 0·4 mg. radioacetate (3 $\mu$ C).

At fixed intervals 3 animals were decapitated and brain, liver and tumour removed. The tissues were hydrolyzed with alcoholic KOH. Cholesterol digitonide and fatty acids were isolated as described.

The *in vivo* experiments were repeated once, giving the same results as those reported.

*Determinations.*

*Cholesterol* was assayed by the colorimetric method of Bloor (1916, 1928); gravimetrical analysis of the digitonide yielded slightly smaller values. The latter were used for calculation of the data. The higher fatty acids were isolated as described and determined gravimetrically.

Determination of the liver *glycogen* as the sum of glucose and glycogen was based upon the method of Mendel, Kemp and Myers (1954).

*Coenzyme A* was assayed by the method of Handschumacher, Mueller and Strong (1951). A coenzyme A preparation purchased from Pabst Laboratory served as a reference. It contained approximately 75 per cent of the reduced form of the coenzyme, equivalent to about 300 Lipmann units per mg. by the method of Kaplan and Lipmann (1948).

*Oestrogens.*—Fresh tumour tissue was lyophilized immediately after removal from the animal and the dry material powdered in a mortar. Continuous extraction of this powder with chloroform for eight hours yielded an extract which was purified and solved in 1·2 ml. olive oil after the removal of the chloroform *in vacuo.* The oily solution was administered to ovariectomized mice in 3 subcutaneous injections over a period of 36 hours and the oestrogenic effect was measured by repeated vaginal smears.

*Radioactivity measurements* were made by standard methods. Counting was done with an end-window Geiger Müller counter. In all cases sufficient counts were made in order to keep the probable error below 5 per cent.

Aliquots of fatty acids and cholesterol digitonide were combusted and the $BaCO_3$ was assayed for radioactivity in order to determine the appropriate conversion factors necessary for the calculations of the incorporation data.

### RESULTS AND DISCUSSION.

### In vitro *Experiments.*

A. *Transplanted tumours of the mouse ovary (granulosa cell and sarcomatoid type).*

Preliminary experiments showed that no radioactivity was detectable in the digitonin precipitable fraction obtained from ovarian tumour T 5438 after incubation of the tumour slices with acetate-1-$C^{14}$ of rather low specific activity ; a small but significant activity was observed in the cholesterol derived from Tumour 5441. However, experiments in which acetate-1-$C^{14}$ of higher specific activity was used demonstrated that Tumour 5438 was capable of synthesizing cholesterol *in vitro*. The data are not reported here because they were obtained under conditions which were not always uniformly chosen. The results revealed a striking difference in the cholesterol content of the two ovarian tumours which were both of the granulosa cell type. The original difference observed in counts/min. of the respective cholesterol fractions was very probably due to the variation in cholesterol content, thereby causing a higher dilution of activity in Tumour 5438 than in Tumour 5441. Tumour 5438 was not further studied in detail. Other tumours of this kind were also found to contain varying amounts of cholesterol, as is shown in Table II.

TABLE II.—*Cholesterol and Fatty Acid Content of Transplanted Ovarian Tumours of the Mouse.*

| Tumour. | Type. | Cholesterol (mg./g. wet weight of tissue). | Long chain fatty acids (mg./g. wet weight of tissue). |
|---|---|---|---|
| T 5438 (g) | Granulosa cell | 6–10 | — |
| T 5441 (g) | Ditto | 4– 5 | 13 |
| T19957 (g) | ,, | 10–15 | 13–15 |
| T26567 (g) | ,, | 6– 7 | 18 |
| T26567 (s) | Sarcomatoid | 2·5 | 11 |
| T24202 (s) | ,, | 2·6 | 16 |

Granulosa cell tumours are known to produce oestrogens (Mühlbock, 1952) and a hormonal effect could always be noted on the secondary sex organs of the host bearing the granulosa cell tumours mentioned in Table II. Among these, Tumour 19957 was outstanding by its high content of oestrogenic material which was readily extractable from the lyophilized tumour tissue. We were unsuccessful however in preparing extracts containing oestrogenic activity from the other tumours.

In repeatedly transplanted carcinomas certain changes in histological structure have long been known and are referred to as sarcomatous transformations. This transformation has also been observed in granulosa cell tumours of the ovary

(van Rijssel *et al.*, 1954) and is accompanied by a faster growth rate of the tumour transplants and a loss of oestrogenic activity.

In contrast to the rather high cholesterol content of the granulosa cell tumours, the sarcomatoid tumours were found to contain a much smaller amount of steroid. The fatty acid content did not show this drop.

TABLE III.—*Biosynthesis of Cholesterol and Fatty Acids from Acetate*-1-*C*-14 *by Slices of Transplanted Granulosa Cell and Sarcomatoid Tumours of the Ovary* (*Mouse*).

| | | Cholesterol. | | Fatty acids. | |
|---|---|---|---|---|---|
| Number of experiments. | Tumour. | Counts/min.* | mmoles acetate incorporated per g. wet weight of tissue. × $10^5$. | Counts/min.* | mmoles acetate incorporated per g. wet weight of tissue. × $10^5$. |
| 14 | T 5441(g) | 1200± 540<br>(400–2400) | 3·8 ±1·7<br>(1·3 –7·6) | 2700±1580<br>(730–6100) | 14·6± 8·5<br>(4·0–33·3) |
| 6 | T 19957(g) | 152± 44<br>(64– 228) | 1·1 ±0·32<br>(0·46–1·65) | 992± 235<br>(640–1360) | 5·8± 1·4<br>(3·7– 7·7) |
| 3 | T 26567(g) | 268± 44<br>(180– 340) | 1·1 ±0·18<br>(0·8 –1·4) | 2426± 623<br>(1522–3346) | 18± 4·5<br>(11·3–24·8) |
| 6 | T 26567(s) | 2408± 840<br>(1428–4476) | 4·2 ±1·5<br>(2·5 –7·8) | 729± 362<br>(308–1482) | 3·4± 1·7<br>(1·5– 6·7) |
| 9 | T 24202(s) | 844± 496<br>(160–2656) | 1·5 ±0·9<br>(0·3 –4·9) | 600± 200<br>(261– 815) | 4·0± 1·3<br>(1·7– 5·4) |

* " Infinitely thick " layers of cholesterol digitonide and fatty acids were plated directly on 1 square centimetre area. The cholesterol data were obtained by multiplying the counts per minute of cholesterol digitonide by four.

The results of the experiments in which the cholestero- and lipogenesis† of these tumours have been studied are given in Table III. Although the granulosa cell (g) type tumours contain more cholesterol than the sarcomatoid (s) type, the rate of cholesterol synthesis on a whole does not differ markedly among the two groups.

On the other hand the (g) type tumours seem to possess a somewhat higher rate of fatty acid synthesis than the (s) type.

In discussing the individual (g) and (s) types, the tumours 26567 lend themselves to a fair comparison since they descend from the same tumour. In this case differences in metabolic activity are pronounced, showing a higher rate of cholesterogenesis and a lower rate of lipogenesis in the (s) type of the tumour.

Applying the results concerning the rates of synthesis and content of cholesterol to *in vivo* conditions the most plausible explanation seems that cholesterol catabolism is, in general, more extensive in the (s) types of the tumours, and especially so in Tumour 26567(s). Because of its high oestrogenic content Tumour 19957 was of special interest and particularly since the highest amount of cholesterol was found in this tumour. However, the rate of cholesterogenesis was definitely not the highest and lipogenesis was of the lowest order among the tumours of the (g) type.

† In order to facilitate description, the terms lipogenesis for fatty acid synthesis, and cholesterogenesis for cholesterol synthesis, have been used.

B. *Hepatomas* (transplanted, spontaneous).

It was found that several hepatomas of various origin may also vary in their synthetic properties.

Data on two transplanted and one spontaneous hepatoma are given in Table IV.

TABLE IV.—*Biosynthesis of Cholesterol and Fatty Acids* in vitro *from Acetate*-1-$C^{14}$ *by Slices of Mouse Hepatomas.*

| | Cholesterol. | | Fatty Acids. | |
|---|---|---|---|---|
| Tumour. | mg./g. wet weight of tissue. | mmoles acetate incorporated per g. wet weight of tissue. $\times 10^5$. | mg./g. wet weight of tissue. | mmoles acetatees incorporated per g. wet weight of tissue. $\times 10^5$. |
| T 15282 . . . . | 3·7 | 35·1 . | 25 | 17·5 |
| | | 35·8 . | | 24·7 |
| | | 33·1 . | | — |
| T 26473 . . . . | 5 | 6·25 . | 50 | 36·4 |
| | | 3·75 . | | 15·0 |
| | | 7·0 . | | 32·6 |
| Spontaneous hepatoma . | 6 | 36·1 . | 50 | 86·1 |
| | | 44·7 . | | 105 |

Although Tumour 15282 was grossly contaminated with necrotic areas, which were impossible to remove, its rate of cholesterogenesis was of the same order as that of liver. At the end of the present experiments, this hepatoma did not " take " on further transplantation. An experiment with a tumour of the last generation at hand revealed a lowering of all synthetic capacities as compared with the three earlier experiments reported in Table IV. The transplanted Hepatoma 26473, which was practically free of necrosis, showed a low rate of cholesterol synthesis. Such a difference was not observed between the syntheses of fatty acids in both tumours. The cholestero- and lipogenesis were pronounced in the spontaneous hepatomas. For obvious reasons these data cannot be compared with the others ; it should be noted that the livers of the hybrids, in which a high incidence of these spontaneous hepatomas was observed (Mühlbock, unpublished communication) contain a large amount of fatty material.

C. *Levels of coenzyme A in the tumours.*

Since acetate is being converted to acetyl coenzyme A, prior to its participation in metabolic reactions, the level of coenzyme A (CoA) might be a rate limiting

TABLE V.—*Coenzyme A Content of Some Mouse Tumours.*

| Tumours. | Number of determinations. | Lipmann units per g. fresh tissue. |
|---|---|---|
| Of the ovary : | | |
| T 5441(g) . . . . | 8 . | 20–30 |
| T26567(s) . . . . | 3 . | 15–22 |
| T24202(s) . . . . | 5 . | 10–15 |
| Hepatomas : | | |
| T15282 . . . . | 4 . | 11–17 |
| T26473 . . . . | 2 . | 20–30 |
| Spontaneous hepatoma . | 2 . | 50–70 |

factor in the biosynthetical reactions studied in the present investigation. Therefore the content of CoA was determined in several of the tumours studied (Table V). The levels found in the transplanted mouse tumours were of the same order as those reported in the literature for a number of rat tumours (Strenght and Seibert, 1954 ; Higgins, Miller, Price and Strong, 1950).

In the group of the transplanted ovarian tumours (g and s type), the CoA content of the tumours appears to broadly parallel their synthetical capacities *in vitro*. On the other hand, a discrepancy in the CoA content and level of syntheses of the Tumours 5441 and 15282 was found. From this it was concluded that, in general, the content of CoA need not be the rate limiting factor in the biosynthetical reactions studied.

A less pronounced drop in the CoA content of the spontaneous as compared with the transplanted hepatomas, exists with respect to the normal liver. The livers of the tumour-bearing animals were also assayed. For the greater part no significant deviations from the values obtained for normal liver (100–150 Lipmann units per gram fresh tissue) were found except in some cases with animals bearing the Tumour 5441 during the longest tolerable period (up to forty-five days). A considerable drop in CoA content was repeatedly noted then.

D. *Livers of normal and tumour-bearing* $F_1$ *(♀ C57 Black × ♂* $DBA_f$*)† mice.*

Mice bearing transplants of a granulosa cell tumour of the ovary develop an increased blood volume and an enlarged and spongy liver (Mühlbock, 1952). The normal liver was found to contain 6·7 per cent glycogen on the average, while its counterpart in the T5441-bearing animals contained a smaller percentage, varying from 2·3–4·4 per cent. The condition and the metabolic activity of the liver of the intact tumour-bearing animal may be of interest since it can be assumed that only part of the cholesterol and fatty acids (Medes, Thomas and Weinhouse, 1953. *cf.* E) is synthesized in the tumour, whereas the remaining is derived from plasma cholesterol and fatty acids which originate from the liver. In this connection the synthetic capacities of the livers of mice bearing T5441-transplants were compared with the livers of the corresponding normal mice (Table VI). Since the glycogen content has been shown to be directly related to the amount of acetate carbon converted to fatty acid by liver slices (Haugaard and Stadie, 1952), a difference between the incorporation of tracer could be expected in these

TABLE VI.—*Biosynthesis of Cholesterol and Fatty Acids* in vitro *from Acetate*-1-$C^{14}$ *by Liver Slices of Normal and T5441-bearing BDz Mice.*

| mmoles acetate incorporated per g. fresh tissue. × $10^5$. | | | |
|---|---|---|---|
| Normal liver. | | Liver of tumour-bearers. | |
| Cholesterol. | Fatty acids. | Cholesterol. | Fatty acids. |
| 30·7 | 40·6 | 12·2 | 24·3 |
| 61·4 | 65·7 | 30·0 | 57·2 |
| 80·3 | 51·3 | 27·9 | 28·6 |
| 24·3 | 47·6 | 39·2 | 22·4 |
| | | 77·5 | 235·0 |
| Mg./g. wet weight. | Mg./g. wet weight. | Mg./g. wet weight. | Mg./g. wet weight. |
| 2·8 | 11 | 3 | 16 |

† abbreviated as BDz.

experiments. Except for one experiment in which an unusually high incorporation was found, the average incorporation of acetate into fatty acids in the remaining five experiments with livers of tumour-bearing animals appears to be on a lower level than the corresponding value of normal liver. Almost the same does relate to the synthesis of cholesterol.

Further studies are needed however to establish a strict relationship between tumour weight, weight, and glycogen content of the livers, on one hand, and the lipogenesis of the liver on the other.

E. In vivo *experiments.*

The labelling of cholesterol and fatty acids in normal and neoplastic tissue of $F_1$ (♀ C57 Black × ♂ $DBA_f$) mice bearing the transplanted ovarian tumour T5441

FIG. 1.—The *in vivo* incorporation of $C^{14}$ into cholesterol of whole liver, tumour and brain of male BDz mice bearing the transplanted ovarian tumour 5441 after an intraperitoneal injection of 0·4 mg. acetate-1-$C^{14}$ (3 μC). Total wet weight: liver 1·5 g.; tumour 3·5 g. Total cholesterol content: liver 4·2 mg.; tumour 16 mg. ○——○ liver; ×——× tumour; ▲——▲ brain.

and of CBA mice bearing the transplanted hepatoma T15282 was studied after an intraperitoneal injection of 3 μC sodium acetate-1-$C^{14}$. In these experiments the incorporation of tracer into cholesterol and fatty acids of the liver, brain and the tumour was followed over both a 2 and a 70-hour period.

Typical results obtained with animals bearing the granulosa cell tumour of the ovary are presented in Fig. 1 and 2 and those with mice bearing the hepatoma in Fig. 3 and 4. In all figures the total amount of acetate incorporated into the respective constituents of the whole organ is plotted against time. The curves thus obtained illustrate a rapid turnover of liver cholesterol and fatty acids in tumour-bearing animals, studied earlier in normal animals by Hevesy, Ruyssen and Beeckmans (1951), Beeckmans, Casier and Hevesy (1951), and Hutchens, van Bruggen and West (1954).

It was not possible to detect any measurable radioactivity in the brain cholesterol under the conditions of the present experiments. As it was felt that the presence of the tumour could possibly drain off radioactive precursors normally reaching the brain, an identical experiment was performed with normal mice of identical genetic constitution. In this experiment also the brain cholesterol was found to contain no radioactivity. Concomitantly with the decrease in total radioactivity of the liver cholesterol, the corresponding activity shown by the cholesterol of the ovarian tumour slowly increased with time, reaching a maximum at about 16 hours. The total activity incorporated into the cholesterol of the tumour tissue (3·5 g. on the average) surpassed that of liver at about 12 hours after administration of the radioacetate and remained approximately constant from 16 hours until the end of the experiment. These data reflect an active cholesterol catabolism in a non-growing tissue, the liver, and little if any catabolic activity in the growing tissue of the tumour T5441. Thus, this tumour can be considered as behaving like a cholesterol " trap ".

FIG. 2.—The *in vivo* incorporation of $C^{14}$ into long chain fatty acids of whole liver, tumour and brain of male BDz mice bearing the transplanted ovarian tumour 5441 after an intraperitoneal injection of 0·4 mg. acetate-1-$C^{14}$ (3 $\mu$C). Total wet weight : liver 1·5 g. ; tumour 3·5 g. Total content of fatty acids : liver 17 mg. ; tumour 20 mg. ; brain 11 mg. ○——○ liver ; ×——× tumour ; ▲——▲ brain.

The time course of cholesterol labelling in liver and tumour demonstrates that $C^{14}$ is transferred from the liver to the tumour area and is stored there in cholesterol. Synthesis *de novo* in the tumour depends on the supply of acetate to the tumour. Although no data are available on the time sequence of acetate disappearance from blood after intraperitoneal injection into tumour-bearing mice, it seems justified, in view of the experiments with rats by Busch (1953) and Busch and Baltrush (1954), to assume that within a very short time (10 minutes) the amount of radioacetate remaining in the blood forms but a very small percentage of the original. To what degree the two processes resulting in the cholesterol labelling of the tumour are operating remains undecided but it appears from what has been said that *total* synthesis will be of importance in the very beginning of the experiment, whereas transfer from the liver will dominate in the later stages. As a consequence the blood supply has its definite bearing on both processes. The ovarian tumour is nearly free of necrosis and the blood supply is rather well organized. This does not hold true for the hepatoma. Notwithstanding this, the total incorporation of tracer into hepatoma cholesterol after 10 minutes is much higher than that into the cholesterol of the ovarian tumour, even though the latter surpasses the former more than twice in weight.

The data of these *in vivo* experiments seem to confirm the *in vitro* results, i.e. the much higher rate of cholesterol synthesis in the hepatoma as compared with that of the ovarian tumour. The fact that cholesterol labelling of the hepatoma

FIG. 3.—The *in vivo* incorporation of $C^{14}$ into cholesterol of whole liver, tumour and brain of female CBA mice bearing the transplanted hepatoma 15282 after an intraperitoneal injection of 0·4 mg. acetate-1-$C^{14}$ (3 $\mu$C). Total wet weight : liver 1·5 g. ; tumour 1·5 g. Total cholesterol content : liver 4·1 mg. ; tumour 5·6 mg. ○——○ liver ; ×——× tumour ; ▲——▲ brain.

FIG. 4.—The *in vivo* incorporation of $C^{14}$ into long chain fatty acids of whole liver, tumour and brain of female CBA mice bearing the transplanted hepatoma 15282 after an intraperitoneal injection of 0·4 mg. acetate-1-$C^{14}$ (3 $\mu$C). Total wet weight : liver 1·5 g. ; tumour 1·5 g. Total content of fatty acids : liver 36 mg. ; tumour 38 mg. ; brain 11 mg. ○——○ liver ; ×——× tumour ; ▲——▲ brain.

never exceeded that of the liver must be partially due to the low mass of the tumours (1·5 g. on the average). In this respect liver and tumour may be compared since they have approximately the same weight. The total incorporation of radioactivity in the cholesterol fraction of this tumour was much like that of the liver, as can be seen in Fig. 3.

The results of the total incorporation of $C^{14}$ into the fatty acid fractions of both tumours and the livers of the corresponding animals are illustrated by Fig. 2 and 4. It appears from this, that the livers of hepatoma-bearing mice incorporate much more of the administered tracer into their fatty acid components than the livers of the ovarian tumour-bearing animals. Apart from the strain difference it should be borne in mind that livers of granulosa cell tumour-bearing animals show an enlarged and spongy appearance. This may have its bearing on the difference in lipogenesis observed.

The total amount of acetate incorporated into the fatty acids of both tumours does not differ markedly.

The authors wish to acknowledge their indebtedness to Dr. O. Mühlbock, Dr. Th. G. van Rijssel and Dra. R. van Nie for many helpful discussions regarding the biological problems inherent in the present investigation and for making available the experimental animals and tumours.

### SUMMARY.

1. The biosynthesis of cholesterol and long-chain fatty acids in surviving tissue slices of several transplanted mouse ovarian tumours of the granulosa cell type, and the sarcomatoid type was studied with acetate-1-$C^{14}$.

2. Granulosa cell tumours of the ovary contained more cholesterol than the sarcomatoid type of these tumours. Among the former group, the tumour with the highest oestrogen titre was found to contain the highest amount of cholesterol but not the highest rate of cholesterogenesis. The granulosa cell and sarcomatoid tumour which descended from the same tumour showed differences in the rates of their respective metabolic reactions leading to cholesterol and fatty acids.

3. Although the coenzyme A content of the tumours was found to parallel their synthetic capacities broadly, the level of CoA need not be the rate limiting factor in the biosynthetic processes studied.

4. The livers of mice bearing a granulosa cell tumour were studied, but no clear-cut conclusions as compared with normal liver could be made.

5. Including the results obtained from identical experiments with three hepatomas, it can be stated in general that a distinct rate of synthesis *in vitro* was not found to be an exclusive property of all tumours belonging to one of the three groups studied.

6. The time course of labelling of the cholesterol and fatty acids in the liver, brain and tumour of intact mice bearing a granulosa cell tumour or hepatoma, was followed after administration of a single dose of acetate-1-$C^{14}$. The *in vivo* data of the hepatoma cholesterol resembled those of the liver, whereas little cholesterol catabolism was found to occur in the granulosa cell tumour. Hepatic lipogenesis differed markedly in the two cases.

### REFERENCES.

Baker, C. G. and Greenberg, D. M.—(1949) *Cancer Res.*, **9**, 701.

Beeckmans, M. L., Casier, H. and Hevesy, G.—(1951) *Arch. int. Pharmacodyn.*, **86**, 33.

Bloor, W. R.—(1916) *J. biol. Chem.*, **24**, 227.—(1928) *Ibid.*, **77**, 53.
Busch, H.—(1953) *Cancer Res.*, **13**, 789.
*Idem* and Baltrush, H. A.—(1954) *Ibid.*, **14**, 448.
de Flines, J.—(1951) *Experientia*, **7**, 234.
Furth, J. and Butterworth, J. S.—(1936) *Amer. J. Cancer*, **28**, 66.
Handschumacher, R. E., Mueller, G. C. and Strong, F. M.—(1951) *J. biol. Chem.*, **189**, 335.
Haugaard, E. S. and Stadie, W. C.—(1952) *Ibid.*, **199**, 741.
Heard, R. D. H., Jacobs, R., O'Donnell, V., Peron, F. G., Saffran, J. C., Solomon, S. S., Thompson, L. M., Willoughby, H. and Yates, C. H.—(1954) *Recent Progr. Hormone Res.*. **9**, 383.
Hevesy, G. Ruyssen, R. and Beeckmans, M. L.—(1951) *Experientia*, **7**, 144.
Higgins, H., Miller, J. A., Price, J. M. and Strong, F. M.—(1950) *Proc. Soc. exp. Biol. N.Y.*, **75**, 462.
Hutchens, T. T., van Bruggen, J. T. and West, E. S.—(1954) *Arch. Biochem. Biophys.*, **52**, 261.
Kaplan, N. O. and Lipmann, F.—(1948) *J. biol. Chem.*, **174**, 37.
Kats, J. and Chaikoff, I. L.—(1954) *Ibid.*, **206**, 887.
Li, M. H. and Gardner, W. U.—(1947) *Cancer Res.*, **7**, 549.
Lieberman, S. and Teich, S.—(1953) *Pharmacol. Rev.*, **5**, 285.
Medes, G., Thomas, A. and Weinhouse, S.—(1953) *Cancer Res.*, **13**, 27.
Mendel, B., Kemp, A. and Myers, D. K.—(1954) *Biochem. J.*, **56**, 639.
Mühlbock, O.—(1952) *Geburtsh. u. Frauenheilk.*, **12**, 289.—(1954) 'Derde Jaarboek van Kankeronderzoek en Kankerbestrijding in Nederland,' p. 103.
Olson, R. E.—(1951) *Cancer Res.*, **11**, 571.
Popják, G.—(1950) *Biochem. J.*, **46**, 560.
van Rijssel, Th. G., van Nie, R., Mühlbock, O. and de Bruyn, W. M.—(1954) 'Vierde Jaarboek van Kankeronderzoek en Kankerbestrijding in Nederland,' p. 17.
van Slyke, D. D., Plazin, J. and Weisiger, J. R.—(1951) *J. biol. Chem.*, **191**, 299.
Strenght, D. R. and Seibert, M. A.—(1954) *Proc. Amer. Ass. Cancer Res.*, **1**, 47.
Zamecnik, P. C., Loftfield, R. B., Stephenson, M. L. and Steele, J. M.—(1951) *Cancer Res.*, **11**, 592.